# Psychosocial therapeutic approaches for high-functioning autistic adults

**DOI:** 10.3389/fpsyt.2023.1265066

**Published:** 2024-01-11

**Authors:** Tina Schweizer, Dominique Endres, Isabel Dziobek, Ludger Tebartz van Elst

**Affiliations:** ^1^Department of Psychiatry and Psychotherapy, Medical Center - University of Freiburg, Faculty of Medicine, University of Freiburg, Freiburg, Germany; ^2^Department of Psychology, Clinical Psychology of Social Interaction, Faculty of Life Sciences, Humboldt-Universität zu Berlin, Berlin, Germany; ^3^Berlin School of Mind and Brain, Humboldt-Universität zu Berlin, Berlin, Germany

**Keywords:** autism, high-functioning, adults, interventions, therapy, treatment, psychosocial, cognitive-behavioral

## Abstract

Autism spectrum disorder (ASD) is characterized by impaired social interaction and communication skills, repetitive behaviors, restricted interests, and specific sensory processing. Particularly, adults with high-functioning ASD often remain unrecognized, presumably due to their high compensatory skills, but at the cost of high stress, which is often linked to anxiety and depression. This may further explain the significantly high suicide rates and reduced life expectancy among individuals with ASD. Thus, providing support to high-functioning autistic adults in managing core symptoms, as well as co-occurring anxiety and depression, appears essential. To date, only a limited number of evidence-based psychosocial therapeutic options are available, and very few of them have undergone rigorous evaluation in a clinical context. To obtain a comprehensive understanding, a systematic literature search was conducted according to the PRISMA checklist, and only studies demonstrating robust methodological quality were included and discussed in this review article. Although promising initial key factors and methods have been identified, additional evidence-based studies are imperative to ascertain the optimal treatment and evaluate the long-term outcomes for adults with high-functioning ASD.

## Introduction

Autism spectrum disorder (ASD) comprises a heterogeneous group of neurodevelopmental disorders characterized by impaired social interaction and communication skills, restricted patterns of behaviors and interests, and specific sensory processing. Within this spectrum, adults with high-functioning autism, characterized by quite normal language use and no intellectual impairment, are often overlooked, even by healthcare professionals, due to their less severe core symptoms. Instead, they may predominantly present co-occurring symptoms, such as anxiety or depression, along with secondary issues like frequent work changes or unemployment (1). However, it is imperative to recognize that these individuals frequently experience significant impairment, which they may compensate for at the cost of enduring elevated stress. The phenomenon of “masking” or “social camouflaging” is prevalent, often leading to a state known as “autistic burnout” (2, 3) and complicating the process of receiving an ASD diagnosis (4). As a result, adults with high-functioning autism often seek psychotherapy to address challenges related to depression, anxiety, difficulties in social interaction and communication, as well as coping with everyday life and stress regulation (5, 6).

Despite the evident need for therapeutic support in this clinical population, their access to appropriate healthcare services remains insufficient. In contrast to the availability of specialized treatments for children and adolescents, few tailored options exist for adults with high-functioning ASD. Additionally, even when in contact with the healthcare system, they often receive less adequate treatment (5, 7). Furthermore, research concerning treatments for autism predominantly focuses on children and adolescents, with limited investigations addressing interventions for adults with ASD. These studies examining treatments for adults with high-functioning autism frequently suffer from small sample sizes, biased samples, absence of control conditions, and lack of randomization. Moreover, the diversity in intervention content, procedures, assessment methods, and outcomes across studies makes it challenging to summarize the findings. Consequently, there remains very limited evidence regarding interventions for high-functioning autistic adults. Nonetheless, both the current National Institute for Health and Clinical Excellence (NICE) (8) and German (6) guidelines advocate psychosocial interventions for autistic adults, particularly regarding the management of core symptoms, co-occurring mental health conditions, daily life skills, and stress.

The most promising psychosocial approaches, to date, are based on Cognitive Behavioral Therapy (CBT), which emphasizes thoughts and beliefs to understand and modify behavior and emotional experiences. The structured nature of CBT, combined with the provision of information about ASD and the therapeutic methods, goal-setting, and home assignments, is well-suited for individuals with autism, corresponding with their need for predictability and information. In addition, the training of specific skills, also in daily situations, might support the translation from specific to more general skills and the generalization across different situations, which is specifically challenging for ASD individuals. Moreover, Mindfulness-Based Interventions, as a more recently developed CBT approach, complement the traditional approaches by focusing on present thoughts, emotions, and perceptions with acceptance and without evaluation. Mindfulness-Based Interventions aim to increase psychological flexibility and potentially reduce anxiety and depression symptoms.

This review aims to systematically outline the current evidence-based psychosocial approaches for treating the most prominent core and associated symptoms in high-functioning autistic adults. Focusing only on well-designed research studies that investigate these interventions with a high level of methodological validity, a systematic search and analysis was conducted according to the PRISMA schema to present, summarize, and discuss the relevant literature on this topic.

## Methods

A targeted literature search was conducted in January 2023 using the databases MEDLINE, PsycINFO, and PsycARTICLES via the EBSCO interface by combining all of the following search terms: (a) “autism spectrum disorder,” “autism,” “autistic” or “ASD,” AND (b) “high-functioning,” “Asperger,” or “without intellectual impairment,” AND (c) “therapy,” “intervention,” or “treatment.” Only original articles written in English and from peer-reviewed journals were considered, supplemented by a search in current national guidelines (e.g., NICE). Additionally, the reference lists of the included articles were screened. Studies targeting adult patient samples (> 18 years) with an IQ > 70 were included, while those with younger or mixed age samples and with an IQ < 70, as well as studies without a description of age or IQ, were excluded. The inclusion of articles was first determined through an evaluation of the title/abstract and, subsequently, the full publication. The literature search was performed by one clinical expert rater.

**Figure 1:**
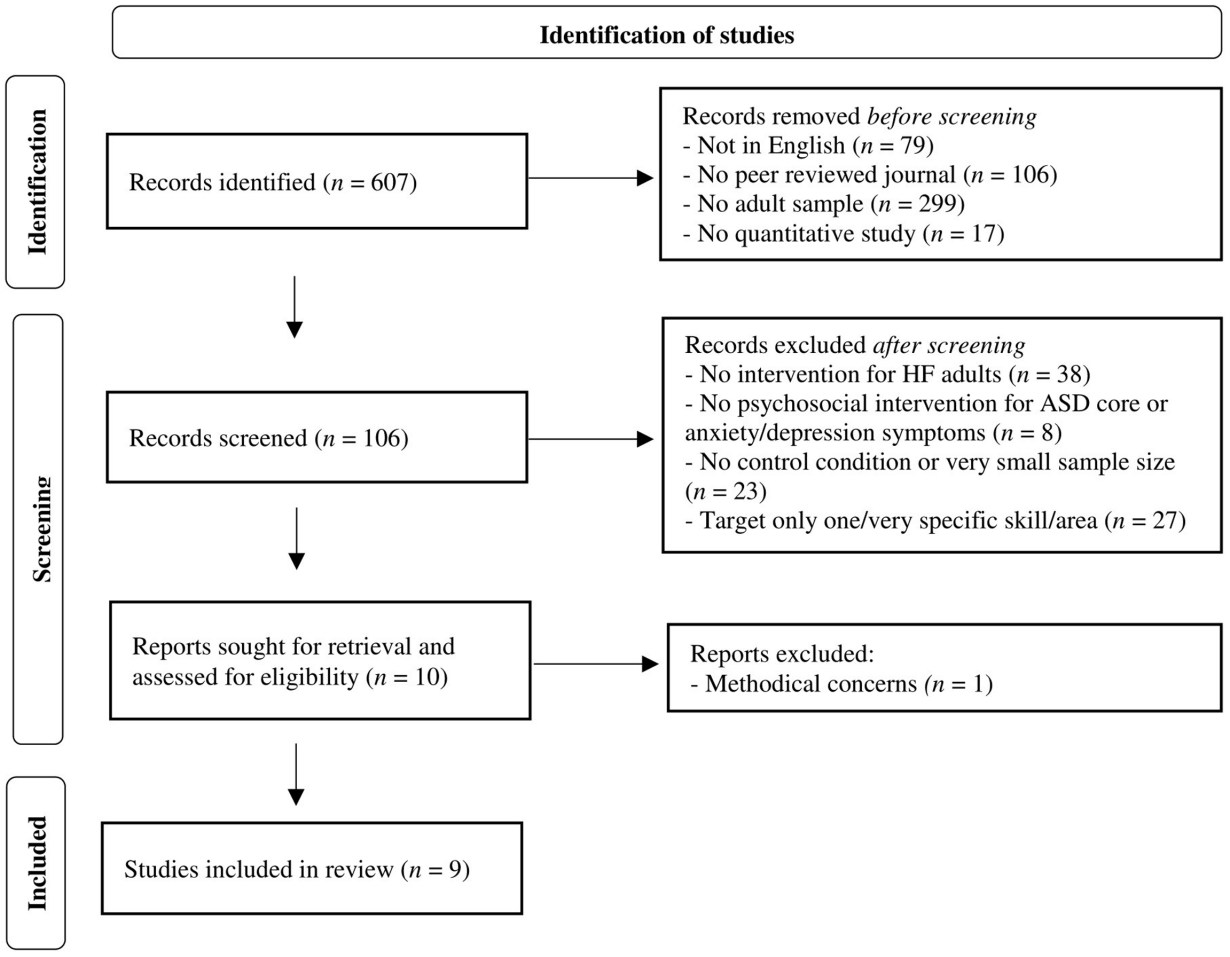
Flowchart of the literature search and selection process.

To evaluate exclusively the evidence-based therapeutic approaches for autistic adults, only quantitative and controlled trials/studies were included. To ensure the minimum degree of statistical power, only studies with a sample size of *N* > 16 were considered, as this is the minimal sample size for detecting a potential large effect between groups (within-between interaction effect of *f* = 0.4, repeated measure ANOVA, power (1-β) = 0.80, α-level = 0.05, one-tailed testing). For the detection of median or small effects, even larger sample sizes would be necessary. With a focus on psychosocial interventions, only interventions targeting core or co-occurring anxiety and depression symptoms of ASD were included, while interventions targeting other comorbid specific disorders (e.g., OCD) or subgroups (e.g., only unemployed individuals) in ASD samples, as well as biological (e.g., TMS) or somatic (e.g., biofeedback, dance therapy) interventions, were excluded. Furthermore, only psychosocial interventions targeting various domains of social functioning were included, while interventions targeting only one specific skill (e.g., emotional face recognition) and/or only very specific skills (e.g., training of one strategy for better reading) or settings (e.g., only employment/academic) were excluded (for an overview of the selection process, see Figure 1, based on the PRISMA schema).

## Results

The keyword search via the EBSCO interface revealed 607 articles, with 528 written in English and 422 published in peer-reviewed journals. After filtering for adult samples (>18 years), 123 articles remained. Further narrowing to quantitative studies revealed 106 articles. After excluding articles related to diagnostics or observational studies, samples containing individuals with intellectual impairment or other intervention populations (e.g., parents of ASD individuals), 68 articles remained, related to interventions for high-functioning autistic adults. After further exclusion of articles with no psychosocial intervention for ASD core, anxiety or depression symptoms (e.g., targeting OCD; biological/somatic interventions), no control condition, sample sizes of N < 16, or interventions targeting only one, very specific skill or area (academic/employment context), or due to methodical concerns, nine articles remained, constituting the basis for synthesis and narrative integration (for an overview, see Figure 1). From these studies, information regarding the design, sample size, aims, interventions (including content, methods, and adaptations to ASD), control conditions, setting, and results were systematically extracted. The results of this review were synthesized and incorporated with the clinical experience of the authors (for an overview of the integrated studies and treatment programs, see Table 1).

**Table 1:** Overview of the integrated studies and treatment programs.

Treatment concept	Study description	Primary outcome targets	Design	Sample size	Duration	Setting	Intervention elements	Results
Interventions targeting ASD core symptoms								
PEERS YA	Social skills Program for the Education and Enrichment of Relationship Skills for Young Adults (PEERS YA; age: 18–23 years) vs. wait list CC (9)	Social skills and knowledge, perspective taking skills	RCT	*N* = 22 *n* = 12 IC, 10 CC	14 weeks	- Group intervention - 9–10 participants - 90 min sessions weekly - 2 therapists - Caregiver support group	- Psychoeducation - Socratic dialogue - Social behavior/rules modelling and interaction tasks (e.g., role plays) - Perspective taking tasks - Structured skills practice by behavioral rehearsals - Performance feedback - Homework	- Increased overall social skills, social skills knowledge, social responsiveness, empathy, get-together frequency - Decreased loneliness after intervention
PEERS YA	Social skills Program for the Education and Enrichment of Relationship Skills for Young Adults (PEERS YA; age: 18–23 years) vs. wait list CC (10)	Social skills and knowledge, perspective taking skills	RCT	*N* = 22 *n* = 12 IC, 10 CC	16 weeks	- Group intervention - 10 participants - 90 min sessions weekly - 2 therapists - Caregiver support group	See Gantman et al. (9)	- Increased overall social skills, social skills knowledge, and social engagement - Reduced ASD symptoms related to social responsiveness after intervention
PEERS YA	Social skills Program for the Education and Enrichment of Relationship Skills for Young Adults (PEERS YA; age: 18–23 years) vs. wait list CC (11)	Social skills and knowledge, perspective taking skills	RCT	*N* = 52 *n* = 29 IC, 23CC	16 weeks	- Group intervention - until 10 participants - 90 min sessions weekly - 2 therapists - Caregiver support group	See Gantman et al. (9)	- Increased social skills knowledge, social behavior and responsiveness, empathy - Decreased social anxiety after intervention
ACCESS	Acquiring Career, Coping, Executive Control, Social Skills program (ACCESS; age: 18–38 years) vs. waitlist CC (12)	- Stress and anxiety coping skills - Self-determination skills - Adaptive and social skills	RCT	*N* = 44 *n* = 29 IC, 15 CC	19 weeks	- Group intervention - 14 participants - 90 min sessions and 3 h vocational activity weekly - 1 therapist - Caregiver support group	- Psychoeducation - Emotion recognition/regulation (e.g., cognitive restructuring, reappraisal) - Behavior modelling (e.g., role plays) - Social skills training - Self-talk strategies regarding self-determination (e.g., goal setting, planning/execution, decision-making, problem-solving, self-advocacy) - Homework	Increased coping self-efficacy, adaptive and self-determination skills after intervention
Structured social skills training	Structured social skills training vs. non-specific social interaction CC (13)	Social skills	RCT	*N* = 19 *n* = 10 IC, 9 CC	16 weeks	- Group intervention - 10 participants - 60 min sessions weekly - 1 therapist	- Emotion recognition and responding - Discussions/exercises regarding social situations (e.g., role plays) - Homework	Increased social cognition and responsiveness, functional impairment after both interventions
Interventions targeting ASD co-occurring symptoms and quality of life								
CBT	Cognitive behavior therapy (CBT) vs. recreational activity CC (14)	Co-occurring mental health-related symptoms	RCT	*N* = 75 *n* = 35 IC, 40 CC	36 weeks	- Group intervention - 6-8 participants - 180 min sessions weekly - 2 therapists	- Psychoeducation - Social skills training (e.g., role-plays) - Goal setting - Behavior analysis - Identification/ Reappraisal of dysfunctional thoughts - Exposure exercises - Homework	- Increased quality of life after both interventions (*d* = 0.31) - No chance in sense of coherence, self-esteem, psychiatric symptoms - Lower drop-out rates and higher subjective improvement, wellbeing, understanding of difficulties, ability to express needs after CBT
MBT	ASD adapted Mindfulness Based Therapy (MBT) vs. wait list CC (15)	Co-occurring anxiety and depression symptoms	RCT	*N* = 42 *n* = 21 IC, 21 CC	9 weeks	- Group intervention - 10-11 participants - 150 min sessions and 4–6 h meditation practice weekly - 2 therapists	- Psychoeducation - Mindfulness-based tasks (e.g., body scan, meditation) - Mindfulness-based Coping - Future planning - Homework	- Reduced anxiety (*d* = 0.76), depression (*d* = 0.78), rumination (*d* = 1.25) - Increased positive affect (*d* = 0.79) after intervention
CBT/MBSR	Cognitive behavior therapy (CBT) vs. Mindfulness Based Stress Reduction (MBSR) intervention (16)	Co-occurring anxiety and depression symptoms	Controlled, not randomized trial	*N* = 59 *n* = 27 CBT, 32 MBSR	13 weeks	- Group intervention - 9-11 participants - 90 min sessions weekly - Number of therapists is not mentioned	CBT: - Psychoeducation - Problem definitions - Emotion regulation/Coping - Challenge of dysfunctional thoughts - Plan/execution of tasks - Future planning - Homework MBSR: See Spek et al. (15)	- Reduced anxiety, depression, rumination, autistic symptoms - Increased global mood after both interventions
Online CBT/Online MBT	Online Cognitive behavior therapy (CBT) vs. Mindfulness Based therapy (MBT) vs. waitlist CC (17)	Co-occurring anxiety symptoms	RCT	*N* = 54 *n* = 16 CBT, 19 MBT, 19 CC	6–8 weeks	- Individual intervention - Self-guided online courses	MBT: - Awareness - Non-judgment attitude by instructed exercises CBT: - Psychoeducation - Anxiety management by instructed exercises	- Reduced anxiety symptoms after both interventions (vs. CC) - No Change in depressive symptoms, daily functioning, wellbeing

IC, intervention condition; CC, control condition.

### Interventions targeting ASD core symptoms

Interventions targeting ASD core symptoms primarily focus on social functioning in group settings to facilitate interactions and shared experiences. In this context, five studies could be identified, with three of them investigating the same program (PEERS-YA).

The Program for the Education and Enrichment of Relationship Skills for Young Adults (PEERS YA) (9–11) is manualized and aimed at supporting friendships and the development of autonomy through social skills training. It covers etiquette for games, sports, dating, verbal and digital conversational skills, perspective-taking skills, conflict resolution, and responding to teasing and bullying. The structured sessions follow a fixed order, starting with psychoeducation, followed by exploration of social rules through Socratic dialogue, modeling, and training of various social interactions through role plays, and perspective-taking tasks. Skills practice is conducted through behavioral rehearsals with feedback and homework. Caregivers of the young adults also receive psychoeducation and didactic instructions for supporting the participants with training and applying skills. Three RCT studies demonstrated that the PEERS YA program significantly increased social skills knowledge, overall social skills, social skills behavior and responsiveness, social engagement and empathy, and decreased social anxiety and loneliness, with some effects maintained after 4 months.

The Acquiring Career, Coping, Executive Control, Social Skills program (ACCESS) is another CBT-based social functioning intervention, aiming to increase skills and beliefs for adult functioning (12). It includes three main modules: (1) stress and anxiety coping skills, (2) self-determination skills, and (3) adaptive and social skills (regarding friendships and social etiquette at work). The program involves structured sessions, psychoeducation, learning to detect and change negative emotional states (e.g., through cognitive restructuring and reappraisal), modeling of behavior (e.g., through role plays), and training of social skills. Additionally, self-talk strategies related to self-determination (e.g., goal setting, planning and execution, decision-making, problem-solving, self-advocacy) are explored and trained. Caregivers of the young adults also receive psychoeducation and didactic instructions to support the participants in applying skills in daily life and increasing their autonomy. As additional part of the intervention, participants engage in a vocational activity for 3 h per week. Results showed significantly increased coping self-efficacy related to a stronger belief in the ability to access social support in coping with stressors, as well as increased adaptive and self-determination skills according to caregiver reports.

To investigate if a manualized structured social skills training is more effective than a non-structured social interaction group, another RCT study was conducted in autistic high-functioning adults by Ashman et al. (13). Both interventions covered themes like communication and language, emotion recognition and responding, family and friendships, employment, and dating. While the social skills training was highly structured and used role plays, discussions, multimedia exercises, and homework to foster social learning, the social group primarily discussed these themes but included also some role plays and regular homework. Results showed equal improvement regarding visual social cognition, social responsiveness, and functional impairment with no significant difference between groups. However, participants seemed to appreciate the higher structured approach in the social skills training, as indicated by higher attendance rates.

Summarizing all described treatments targeting social dysfunction as a core symptom of ASD, both the PEERS and ACCESS program are specifically designed to facilitate the transition to adulthood in young high-functioning autistic adults. The included studies demonstrate significant improvements in social functioning related to social skills knowledge and application, social interactions and engagement, empathy, social anxiety and loneliness (9–11), social responsiveness (9–11, 13), and social cognition (13), with a maintenance effect regarding social skills knowledge and application, social engagement and symptoms related to social responsiveness at 4-month follow-up (10). Furthermore, findings showed improved functional impairment, coping self-efficacy and adaptive and self-determination skills (12). However, a non-structured social interaction group, was found to be equally effective in improving social cognition, responsiveness, and functional impairment (13). The duration of the final versions of the interventions ranged from 16 to 19 weeks, with weekly 60–90-min sessions for 10–14 participants per group and, in part, a caregiver-group, in parallel (10–12). The ACCESS program additionally implemented 3 h of vocational activity per week for the participants. While the PEERS program was led by two facilitators (10, 11), the other interventions were led by only one facilitator (12, 13). Adaptations to the needs of autistic adults were integrated by providing a small group format, concrete rules and steps, context and structured practice with feedback, and themes conceptualized according to the needs of young autistic adults (9–11). The ACCESS program was adapted with a focus on high structure, multimodal teaching methods, and concrete activities to ground abstract concepts (12). For the intervention investigated by Ashman, no adaptions were reported (13). To promote generalization, all of the social functioning interventions described include homework tasks (9–13), while both transition-to-adulthood interventions involve caregiver support in the application and training of skills (9–12). While the PEERS program includes further CBT elements, such as frequent practice of skills through role plays and behavioral rehearsals in various contexts (9–11), the ACCESS program addresses the issue of generalizability by providing training in various situations and incorporating a vocational activity for 3 h per week (12).

### Interventions targeting ASD co-occurring symptoms and quality of life

Interventions targeting frequently co-occurring anxiety and depression symptoms in ASD adapt established and well-assessed general CBT approaches to the needs of autistic adults. In this context, four studies could be identified.

One study by Hesselmark et al. (14) compared a CBT intervention with a recreational activity control group on mental health-related symptoms in autistic adults. Both conditions were implemented in highly structured sessions. The manualized CBT intervention contains three modules: (a) self-esteem and ASD awareness, (b) social contacts and handling everyday life, and (c) psychological and physical health, while the recreational condition includes conducting collective recreational activities. Accordingly, the CBT condition involves psychoeducation, training of social skills (e.g., through role plays), goal setting, behavior analysis, identification and reappraisal of dysfunctional thoughts, and related exposure exercises. Results revealed improved quality of life after both conditions and no significant intervention effect for sense of coherence, self-esteem, and psychiatric symptoms. However, CBT was related to lower drop-out rates, higher subjective improvement after intervention, increased wellbeing, understanding of their difficulties, and ability to express needs at follow-up.

Another study by Spek et al. (15) compared Mindfulness-Based Therapy (MBT) with a waitlist control group regarding co-occurring symptoms of anxiety and depression in autistic adults. The intervention aiming to increase own perception and foster acceptance through mindfulness-based tasks, partly related to stress and coping. It involves psychoeducation, practicing body scan, mindful breathing, movement and eating exercises, meditation, evaluation of experiences and training of mindfulness coping strategies, exercise planning, and homework. Results demonstrate a significant decline in anxiety, depression, and rumination, as well as increased positive affect after intervention.

Another study aiming to primarily treat co-occurring symptoms of anxiety and depression compared Mindfulness-Based Stress Reduction (MBSR) and CBT interventions in autistic adults by Sizoo and Kuiper (16). The same MBSR protocol (with minor adaptions) was used as in the study from Spek et al. (15). The CBT intervention covers themes like processing styles, relationships and interactions between thoughts, feelings, and behavior, the cognitive model, and dysfunctional thoughts. Additionally, individual problems, social interaction, signs of stress, coping with stress and negative emotions, and future plans with possible obstacles were addressed. The means of choice were providing information and psychoeducation, problem definitions, reflection, and discussions. Additionally, challenging thoughts, detecting stress, training to cope with stress/negative emotions and of social interaction, reflecting on obstacles for future plans and homework were utilized in a highly structured setting. Results showed equally effective interventions, with significantly reduced anxiety, depression, rumination, and autistic symptoms, as well as increased global mood after both interventions, maintained at the 3-month follow-up.

In a more recent study, conducted by Gaigg et al. (17), CBT and MBT were compared to a neutral control condition regarding anxiety in an online setting (without a therapist) in autistic adults. The MBT intervention included awareness of the present moment and a non-judgmental attitude regarding thoughts and feelings through instructed exercises, while the CBT intervention contained psychoeducation regarding anxiety and anxiety management through instructed exercises. Results showed significantly reduced anxiety after the online MBT and CBT interventions vs. the control condition, as well as maintenance effects at 3- and partly at 6-month follow-up. No intervention effect was found regarding depressive symptoms, daily functioning, and wellbeing.

Summarizing all the described treatments targeting co-occurring ASD symptoms, the integrated studies demonstrated significant improvements in quality of life after a CBT and recreational activity intervention (14). CBT was additionally associated with higher subjective improvement, wellbeing, understanding of difficulties, ability to express needs, and lower drop-out rates. Furthermore, a MBT intervention (15) led to reduced anxiety, depressiveness, and rumination, as well as increased positive affect compared to a control condition. Likewise, a MBSR and CBT intervention (16) showed equal improvements regarding anxiety, depression, rumination, autistic symptoms, and global mood, with a maintaining effect. Regarding online interventions (17), both CBT and MBT led to less anxiety compared to a control condition, even over time, but no intervention effect was found for depressive symptoms, daily functioning, and wellbeing. A maintaining intervention effect was observed for anxiety symptoms at 3-month (16, 17) and somewhat attenuated at 6-month follow-up (17) and for depressive symptoms, only in one study, at 3-month follow-up (16). The duration of the on-site group interventions ranged from 9 to 36 weeks, with weekly 90–180-min sessions for 6–10 participants per group. Notably, one mindfulness-based intervention (15) integrated additionally 4–6 h of weekly meditation practice as homework. All on-site intervention groups were led by two therapists. The self-guided MBT/CBT online courses (17) was implemented with a duration of 6–8 weeks. In one CBT intervention (14) the settings were tailored and included more limit setting/rules and fewer exposure tasks, while in one mindfulness-based intervention (15) breathing exercises and the program duration were extended, cognitive elements were omitted, and supported homework planning were included. All on-site interventions were adapted to the needs of autistic adults by implementing clear language use, including avoiding metaphors (14–16) and, in part, ambiguous or imagination-related language (15). In the on-site CBT vs. mindfulness-based intervention (16) study, both protocols were specifically designed for autistic adults, including features such as a slower pace, descriptions of autism from other autistic adults, instructed repetitions, and supported homework planning. For the self-guided MBT and CBT online courses (17), no specific adaptions to autistic needs were reported. All on-site group-interventions included elements to promote generalization by including homework tasks (14–16) and, in part, supported planning of mindfulness-based exercises in daily life (15, 16), conceptualizing future plans and reflecting on possible obstacles (16).

## Discussion

The present review offers a concise examination and description of the current state of the very few evidence-based psychosocial interventions for high-functioning autistic adults, with a particular focus on CBT approaches including MBT. The findings from the integrated studies provide valuable insights into possible key elements of the interventions and reveal promising preliminary evidence for CBT-based approaches in treating social functioning and co-occurring symptoms of anxiety and depression, as well as enhancing quality of life in autistic adults. The review further supports the importance of adapting interventions to the needs of autistic adults. However, the discussion also underscores the need for further research and improvements in study designs to advance the field of interventions for autistic adults.

### Interventions targeting ASD core symptoms

One of the key findings from the reviewed studies is the significant improvement in social functioning, including social skills, interactions, responsiveness, and empathy (9–11, 13), after CBT-based interventions. These findings highlight the potential of CBT-based approaches to enhance dealing with social interactions by incorporating concrete activities to ground abstract concepts and improve the understanding of social rules and interaction for autistic adults. Additionally, CBT interventions showed positive effects on social cognition, further emphasizing their relevance in addressing core symptoms of ASD. Most of these gains were maintained after the interventions, indicating potential sustainability (10). The CBT interventions also proved effective in reducing social anxiety and feelings of loneliness (9–11), potentially alleviating some of the social challenges commonly experienced by individuals with autism. Moreover, these interventions led to a significant increase in functional abilities and adaptive behaviors (12), which may directly impacts individuals’ ability to navigate daily life and engage in various activities effectively. The increase in coping self-efficacy suggests that participants felt more confident in managing challenges and stressors, contributing to better resilience and overall wellbeing. While these positive outcomes may primarily be attributed to the implemented themes and methods, a higher degree of structure appears to be conducive for attendance in the interventions (13).

Particularly, the PEERS YA program stands out, as to date the best evaluated and effective CBT-based intervention in this regard (9–11). Additionally, the ACCESS program showed promising results (12). Both interventions used a CBT multimodal and high structured approach and implemented themes and methods that might be crucial for their effectiveness. Likewise, adaptations were made according to the needs of autistic adults, incorporating a concrete, highly structured approach and elements that facilitate generalization by enhancing skills application in real-life situations. These adaptations are in accordance with current NICE and German AWMF guidelines recommendations (6, 18, 19). However, further studies are needed to identify the specific elements that lead to the improvements in social functioning and determine the most effective degree of structure (13).

### Interventions targeting co-occurring symptoms and quality of life

Furthermore, the integrated interventions targeting co-occurring symptoms of anxiety and depression and quality of life in high-functional autistic adults showed positive outcomes, indicating the potential value in alleviating these symptoms in this population. The integrated interventions provided preliminary evidence for CBT-based approaches with significant improvements in symptoms of anxiety (15–17) and depression (15, 16) as well as health related factors (14–16). Thus, autistic adults can benefit from both interventions. Both, CBT and MB-based interventions demonstrated similar effectiveness in treating co-occurring symptoms of anxiety (16, 17), mainly with a maintaining effect (16, 17). The results regarding interventions targeting co-occurring depressive symptoms appeared less consistent, but showing also comparable effectiveness in reducing depressive symptoms for CBT and MB-based interventions, with only in part a maintaining effect (14, 16, 17). In the study comparing an CBT and MB-based intervention (16), including an additional passive control condition may have helped ascertain that the improvements were indeed the result of the intervention, rather than being influenced by other factors (even if previous studies have already made that comparison). Furthermore, this study would have benefited from a randomized allocation and/or matching regarding important characteristics, even if participants’ allocation seemed to have happened in an unbiased manner. To date, further well-designed RCTs are needed to elucidate more specifically the relationship between the integrated intervention elements and their effects and further optimize the interventions.

Moreover, the included CBT and MB-based interventions led to decreased rumination and increased positive affect/global mood (15, 16), which are important mental health-related outcomes itself as well as potentially mediating factors for anxiety and depression symptoms. Additionally, findings from the integrated studies demonstrated that quality of life, were comparable positively impacted by CBT and a recreational activity group intervention (14). However, the CBT intervention showed additional benefits, including higher subjective improvement, increased wellbeing, improved understanding of difficulties and ability to express needs, and lower drop-out rates, suggesting CBT may be more effective in addressing the specific needs and challenges faced by individuals with autism. The similar improvement in quality of life might be explained by the broadly similar elements of the highly structured group setting and the resources-oriented and activating content in both conditions. Thus, future studies will have to elucidate, which elements are effective and in what way.

Based on the findings of the reviewed studies, online interventions showed promise in addressing co-occurring symptoms in high-functioning autistic adults. Notably, both CBT and MBT interventions delivered in a cost-effective online setting (17), resulted in reduced anxiety levels in comparison to the control condition. However, neither intervention demonstrated a discernible effect on depressive symptoms, daily functioning, or overall wellbeing. The maintaining effect observed for anxiety symptoms at the two follow-ups underscores the potential enduring benefits of these interventions in mitigating anxiety-related symptoms in high-functioning autistic adults, in an easily accessible and cost-effective online setting. Nonetheless, these preliminary findings should be interpreted with caution due to variations in baseline anxiety levels before intervention, which may impede direct comparison of subsequent changes brought about by the interventions. While these outcomes hold promise, it is imperative for future studies to validate this result through ensuring comparable anxiety levels in all conditions prior to the intervention as well as to compare on-site vs. online interventions and explore the currently lacking research on single versus group-based interventions.

All described on-site treatments targeting co-occurring ASD symptoms showed a similar structure as well as longer duration compared to the online intervention. Potentially, incorporating extended practice might be beneficial to enhance treatment outcomes. Notably, all on-site interventions incorporated a clear language use and elements supporting generalization to cater to the needs of autistic adults. The adoptions made in the reviewed studies are in accordance with the current NICE and German AWMF guidelines (6, 8). However, it is essential to acknowledge that the guidelines are also based on the limited existing studies and expert consensus. Therefore, further research is still needed to strengthen the evidence base.

### Overall assessment of the current evidence base

The present review provides preliminary evidence for CBT-based approaches in addressing social functioning and co-occurring symptoms in high-functioning autistic adults. However, it is important to acknowledge the limitations and gaps in the existing literature. Despite the integrated studies, there remains a shortage of rigorously conducted RCTs with too small sample sizes. Hence, besides the integrated evidence-based studies, future research should focus on rigorously conducting well-controlled RCTs with larger and more diverse samples to establish stronger evidence and provide a more comprehensive understanding for intervention effectiveness. This is particularly crucial considering that the prevalence of ASD is comparable to that of other mental disorders, such as eating or panic disorders, for which multiple well-investigated treatments already exist.

Although the integrated studies have the highest level of evidence-base to date, some minor methodological limitations were identified that should be discussed and addressed in future research. The main limitation of the design of the review is the applied high methodological rigor criteria, narrowing the inclusion of otherwise potentially promising studies. However, these rigorous inclusion criteria are also a strength, ensuring to include only high quality studies. Even if we have carefully chosen the search interface, including international databases of medicine, psychiatry and psychology, using additional databases and including also publications in other languages than English, might have revealed further studies.

A major part of the excluded studies suffer from the lack of control conditions and small sample sizes. While the sample sizes of the included studies were larger compared to most of the existing studies, thus ensuring the detection of large effects, the importance of larger sample sizes for identifying potential medium or small effects, which may still hold clinical relevance, cannot be overstated. To detect the full range of potential effects, the need for larger sample sizes in future studies targeting interventions for autistic adults becomes apparent. By enhancing the sample sizes in future studies, we can gain a more valid understanding of the intervention effects and improve the precision of the findings, ultimately contributing to the advancement of evidence-based treatments for autistic adults.

Furthermore, most of the integrated studies (9, 11–15) relied solely on pre-post measures, with only a few studies including additional follow-up assessments (10, 16, 17) with rather short time frames. Hence, our review emphasizes the importance of investigating the sustainability of intervention effects over longer time periods in future research.

While the inclusion criteria of this review focused on studies with a control group design and mainly integrated RCTs, some methodological limitations still persisted. Studies utilizing solely passive control conditions (9–12, 15) may underestimate the inherent impact of any active component, whereas studies employing solely active control conditions (13, 14, 16) might limit the ability to distinguish specific intervention effects, particularly if the inserted contents and methods are similar (13, 14, 16). By examining both types of control condition (17), a deeper understanding of intervention effectiveness can be gained, facilitating the identification of impactful components and their application in designing more effective and cost-efficient treatments. Such knowledge seems pivotal in tailoring interventions to the specific needs of individuals with autism, where some may benefit from more general factors, like a structured environment and social interaction, while others may require more targeted therapeutic approaches. This may also result in cost reductions for the healthcare system and increase the range of available interventions for autistic adults.

In line with the general research situation, the integrated studies demonstrated a gender imbalance, with a greater proportion of male participants in the included studies. Additionally, many interventions focused primarily on young adult samples (9–12) or individuals of younger or middle-aged age groups. Consequently, the generalizability of the findings to female autistic adults or older age groups is limited. Therefore, future studies should strive to include more diverse samples and address the needs of middle-aged or older autistic adults, especially in the context of social functioning interventions.

Another critical point in the current research situation, which is partly also applicable to the integrated studies, is that the integration of multiple intervention components and outcomes, as well as the utilization of diverse methods, hampers the ability to compare the interventions or summarize their effects. Future research studies could benefit by initially identifying the effective components responsible for intervention effects and subsequently using the same standardized treatment manuals based on these components for implementation. This approach may enhance the consistency and replicability of interventions in future studies.

Regarding the setting, the reviewed studies highlighted the potential of online-based interventions to increase accessibility and reduce barriers to treatment for autistic adults. This is especially important in regions with limited access to services or during times of pandemics, as online interventions may provide a cost-effective and convenient alternative. Particularly, individuals with ASD are often overwhelmed by the demands of daily life, making online-based treatments potentially less stressful and more adaptable to their schedules. Additionally, their high affinity for digital tools may increase the acceptance of online interventions. However, it is imperative to conduct further investigations to validate the efficacy of online interventions and understand their specific benefits and limitations.

As research progresses, there is a growing interest in targeting mental health-related outcomes, such as quality of life, and examining potential mediating factors, such as emotion regulation and coping with negative emotions and stress. The significance of supporting autistic adults in managing daily life stress cannot be underestimated, as it is linked to their social functioning (5, 20, 21). Moreover, addressing dysfunctional coping strategies and emotion dysregulation can have far-reaching implications for quality of life (22), social functioning (23), and co-occurring symptoms (24). Consequently, interventions focusing on emotion regulation and stress management are emerging as essential to improve the quality of life in high-functional autistic adults. Several RCTs are already underway to further investigate these mental health-related factors using CBT approaches in high-functioning autistic adults (25, 26).

As there is a pressing need for well-controlled studies with larger sample sizes to address the specific challenges faced by high-functioning autistic adults and more online-based investigations are needed, our research team is currently evaluating an online group-based social functioning vs. a social cognition training intervention vs. TAU condition in a RCT with a total sample size of *N* = 360 (27). To our knowledge this is currently the world’s largest well-controlled psychotherapy trial in adult ASD. Both interventions are manualized and underwent initial evaluations regarding their feasibility and efficacy. The group intervention comprises modules such as psychoeducation, stress management, and social communication training (article is in the process of publishing), while the computer-based training (28) target social cognition by multimedia materials depicting emotional expressions and complex real-life social situations. Through this rigorous and well-powered RCT trial, we are confident that our research will contribute valuable evidence-based findings toward establishing effective psychosocial interventions for high-functioning autistic adults. The comparison of these two promising interventions may shed light on their relative merits, offering valuable insights into best practices for supporting this population in their social functioning and cognitive skills. Furthermore, this research addresses the growing need for online-based interventions, which may offer increased accessibility and flexibility for individuals with autism.

However, besides addressing only individual factors in autistic adults, it is crucial to consider the adaptation of their environment to better meet their specific needs. Modifications in the sensory, physical, and social surroundings can play a significant role in improving the overall wellbeing and functioning of individuals with autism. This approach aligns with the social model of disability (29), which posits that disability arises from a mismatch between an individual’s needs and the support provided by their environment. To enhance the accessibility and effectiveness of services for autistic adults, adjustments should also be implemented within the healthcare system. Current research recommends creating low-stimulus environments and ensuring communication in the preferred style of autistic adults. Moreover, it is essential to enhance healthcare providers’ knowledge and experience regarding autism and ASD interventions (30). By cultivating a deeper understanding of autism-related challenges and needs, healthcare professionals can better serve and support individuals with autism. Autistic adults themselves have expressed preferences in how they wish to be treated by therapists: They value adjusted communication and expect therapists to possess a profound understanding of autism-specific issues, in addition to demonstrating attentiveness, kindness, and acceptance (6). Integrating these perspectives into research and interventions can lead to more effective and tailored support for individuals with autism, empowering them to achieve their full potential. In conclusion, addressing the environmental factors and accommodating the unique needs of autistic adults alongside individual-focused interventions can significantly contribute to their overall wellbeing and quality of life. By adopting a person-centered approach and aligning with the preferences and perspectives of individuals with autism, we can foster a more inclusive and supportive environment, leading to better outcomes in clinical practice and research endeavors.

## Conclusion

In conclusion, this review serves as a foundation for advancing interventions for high-functioning autistic adults, highlighting the potential of evidence-based CBT-based psychosocial interventions, as well as the need for further research and adaptations to cater to the unique needs of this population. By continuing to address methodological limitations and focusing on evidence-based practices, researchers can contribute to support high-functioning autistic adults. The results of this review highlight the necessity for additional research aimed at developing and validating psychosocial interventions tailored to high-functioning autistic adults, addressing core symptoms as well as co-occurring conditions, while also emphasizing procedures that promote generalization and long-term effectiveness. Specifically, methodologically rigorous randomized controlled trials (RCTs) with sufficient sample sizes are required to further investigate the comparative effectiveness and acceptability of ASD-adapted CBT-based approaches. These investigations should also identify the necessary adaptations of standard procedures from established CBT interventions for treating anxiety and depression symptoms that are most effective and beneficial for the autistic needs. The results of this review indicate a pressing need for more evidence-based interventions to cater to this underserved population effectively. By addressing these research gaps, we can contribute significantly to the improvement of mental health and quality of life in high-functioning autistic adults, ultimately fostering a more inclusive and understanding society.

## Data availability statement

The original contributions presented in the study are included in the article/supplementary material, further inquiries can be directed to the corresponding author.

## Author contributions

TS: Conceptualization, Data curation, Formal analysis, Methodology, Visualization, Writing – original draft, Writing – review & editing. DE: Supervision, Writing – review & editing. ID: Supervision, Writing – review & editing. LT: Conceptualization, Supervision, Writing – review & editing.
